# The Book World of Medicine and Science

**Published:** 1907-02-23

**Authors:** 


### The Book World of Medicine and Science.

The Essentials of Histology, Descriptive and Practical, for the Use of Students. By E. A. Schäfer, LL.D., Sc.D., F.R.S., Professor of Physiology in the University of Edinburgh; formerly Jodrell Professor of Physiology in University College, London. (London: Longmans, Green and Co. 8vo. Price 10s. 6d. net.)

This is the seventh edition of Professor Schäfer's well-known handbook on histology. Seeing that the first edition was published so recently as 1885, it is obvious that the book is in demand and supplies a want. It is probably the best publication upon the subject, and it is indispensable to the medical student. There is no change in the general arrangement of the subject-matter. The whole course of histology is divided into fifty lessons, each lesson constituting a chapter, beginning with instructions how to prepare and examine each tissue, and ending with a full description of the appearances to be seen in the preparations made. Notwithstanding the fact that there are 507 pages of print, the book is by no means cumbersome; it is barely an inch thick. The present edition is enlarged partly by additions to the text, and partly by the provision of new illustrations. The chief addition to the text has been in connection with the central nervous system, the finer structure of which is well described; and the author has been fortunate in being able to reproduce some of Professor Sobotta's figures and a large number of original drawings by Professor Ramon y Cajal. There are altogether 547 figures in the book, almost all of them extremely good and helpful to the student. A new feature is the printing of many of the illustrations in colour, which affords a better idea of the appearance of the stained preparations. When almost everything is good it is a pity that anything not up tc standard was allowed to pass. Excellent though most of the illustrations are, some of them are quite poor. There is no picture in the book which conveys any clear idea of the microscopic appearances of a lymph gland. Fig. 54 may be pardoned for being a teased preparation, but fig. 253 has no such excuse. No student could be expected to learn to recognise lymphatic gland tissue from it. Were it not for the description in the text, it might be taken for a developing bone. Fig. 86, again, professes to represent elastic fibres in cross-section, but it might be a piece of adipo-fibrous tissue; it gives no idea of the sharp angularity that elastic fibres in cross-section present. Again, reproductions of micro-photographs are excellent in their way, but we cannot help feeling that the illustrations of sections of the medulla oblongata and pons would have been much more helpful to the student had they been rendered at least semi-diagrammatic, whilst yet preserving the general characters of an actual section. We are surprised that so large a picture of Oliver's apparatus for estimating blood-corpuscles is given, whilst the Thoma-Zeiss hæmocytometer is not illustrated, and is described in its old form, the directions being given with so little detail that it would be very difficult for a student, working by the book alone without an instructor, to follow them. In the preparation of hæmin crystals no direction is given as to the addition of a minute crystal of sodium iodide as well as glacial acetic acid to the blood, though the addition of such a crystal makes the preparation so much easier. In an appendix directions are given for hardening and embedding various tissues for section, together with an account of microtomes and of special methods. Any account of such methods is of little use unless exact details are given. In a book intended to instruct those who do not already know, it is of little use to give directions such as the following for embedding in paraffin: "Before being soaked in melted paraffin, the piece of tissue may be stained in bulk; it is then dehydrated by a series of alcohols (50 per cent., 75 per cent., 95 per cent.), finishing up with absolute alcohol; after which it is soaked in cedar wood oil, xylol, or chloroform. It is now transferred to molten paraffin, which should not be too hot." The student will at once need to ask someone how long to leave the tissue in each strength of alcohol, how hot the paraffin should be, and so on. We should like to see points like these elaborated, or else the imperfect directions altogether omitted. Though we find fault with some few matters of this kind, we consider the new edition of "The Essentials of Histology" to be, upon the whole, extremely good. It might be even better, but without doubt it will continue to hold the position it has established of being *the* book for general use in the histological laboratory.

The British Journal of Tuberculosis. Edited by T. N. Kelynack, M.D. (London: Baillière, Tindall, and Cox. Vol. I., No. 1. January 1907.)

No one, we think, will be disposed to question a policy which has expressed itself in the resolution to establish a journal devoted to the study of tuberculosis and to the problems which arise in connection with that disease. These problems are in part pathological and so fall within the purview of the medical profession. There are others, however, which are mainly social and economic, and these concern many interests in the general community. The new journal proposes to address itself to all aspects of the subject, and in the first issue may be found fair promise of its ability to achieve this ambitious programme. The Editor has certainly been fortunate in his contributors, and if he is able to maintain the level reached in his first number he certainly will not lack readers. Most effective under the title of "The Study of Tuberculosis" is the "Retrospect" by Professor Clifford Allbutt, and "An Anticipation" by Dr. R. W. Philip. The former has all the grace and charm invariably associated with Professor Allbutt's literary work, and may be specially commended to those who entertain doubts regarding the progress of medical treatment. Dr. Philip discusses modern methods in the light of his own considerable experience, and it is of importance to take notice of his conviction of the great value of tuberculin as a curative agent. Sir Richard Douglas Powell, Sir John Moore, and Dr. Byrom Bramwell contribute articles on the care and control of the consumptive poor, and from these may be gathered some indication of the numerous questions which are raised by the problem of tuberculosis. Other articles are by Sir Lauder Brunton, Sir Hermann Weber, and Sir Samuel Wilks—names which are a guarantee of interesting and authoritative writing. Finally may be mentioned notices of individual sanatoria and health stations, as affording information useful both to patients and practitioners. Altogether the new journal has made an excellent start, and we wish it a successful career. That it will be conducted with judgment and discretion, and will be preserved from the isolation and detachment which are the special dangers of publications appealing to special and peculiar interests, we have no doubt. This is the professed aim of the editor, and every confidence may be placed in his resolution.

### BOOKS RECEIVED.

Baillière, Tindall, and Cox.

